# Measures to Maintain a SARS-CoV-2 Negative Inpatient Hematological Unit in the Midst of the COVID-19 Pandemic

**DOI:** 10.3389/fmed.2020.00462

**Published:** 2020-08-26

**Authors:** Almudena Cabero-Martínez, Fermín Sánchez-Guijo, Lucía López-Corral, Estefanía Pérez, Alejandro Avendaño, Mónica Baile, Mónica Cabrero, Ana-Africa Martín, Angela Rodríguez, Balbina Pérez, Felipe Peña-Muñoz, Luz-Gema Román, Danylo Palomino, Lourdes López-Vázquez, María-Belén Vidriales, Marcos González-Diaz, María-Victoria Mateos, María-Dolores Caballero

**Affiliations:** Hematology Department, University Hospital of Salamanca (HUS/IBSAL), CIBERONC and Center for Cancer Research-IBMCC (USAL-CSIC), Salamanca, Spain

**Keywords:** COVID-19, hematology, preventive measurements, inpatient units, immunodepressed patients, pandemic (COVID-19)

## Abstract

The University Hospital of Salamanca, in Spain, had its first COVID-19 case on March 1st and as of May 11th, we had 1,100 positive cases. Based on the vulnerability of patients with blood cancers, on March 9th, the Hematology Department developed a protocol, amended as the new information was available, to maintain the Hematology Unit as a “free COVID-19 island.” The protocol included symptom-based surveys and screening tests to patients, caregivers, and healthcare personnel to identify early potential positive cases and prevent its spread. Between March 9 and April 28, 32 asymptomatic patients and caregivers were tested and 68 rT-PCR diagnostic assays have been performed with two positive results. A 106 healthcare workers have been tested (208 rT-PCR) and seven of them were positive. In summary, the implementation of preemptive measures after the first case appeared allowed us to be able to provide treatment to our patients.

## Introduction

The first officially reported case of Coronavirus disease 2019 (COVID-19) in Spain was on January 31, 2020, when a German tourist tested positive for SARS-CoV-2 in La Gomera, Canary Island. In February 9, the second case involved a British tourist in Palma de Mallorca, Balearic Island and the third-one, the first death in Spain, affected a 69-years-old man who had been in Nepal, died in Valencia and was diagnosed post-mortem (1, 2).

In early March, an outbreak was identified and on March 13, the Spanish Government declared a National State of Alarm which halted all non-essential activities between March 29 and April 12. From April 13, a lifting of some restrictions occurred although most of them have been extended until May 9. At the time of writing this manuscript, a de-escalation plan in various phases is beginning to be developed by areas.

As of May 11th, 2020, there have been 227,436 confirmed cases in Spain, with 137,139 recoveries and 27,744 deaths in Spain (https://www.mscbs.gob.es/en/progesionales/saludPublica/ccayes/alertasActual/nCov-China/situacionActual.html).

The real number of cases, however, is likely to be much higher, as many people with only mild or no symptoms are unlikely to have been PCR or serology-tested.

The University Hospital of Salamanca, had its first case admitted on March 1st and as of May 11th, 1,584 patients had been admitted in COVID positive Units, most of them with pneumonia and in 1,100 of them, rT-PCR test for SARS-CoV-2 resulted positive.

At the Hematology Department, the concern for in and outpatients with hematological diseases as well as those recipients of autologous/allogeneic hematopoietic stem cell transplantation (HSCT), or CAR-T cells was present since the beginning of the outbreak because of two reasons: (i) we considered our patients were at potential higher risk if they were infected from SARS-CoV-2 because of their vulnerability (3) and (ii) treatment was not possible to be delayed in some patients and we had to balance between providing appropriate treatment at and the possible risk of becoming infected (4).

The main objective of our clinical team since pandemic started was to maintain our unit as negative for COVID-19 trying to identify early potential infections as well as prevent its spread as soon as possible while providing treatment to our patients in need, as safely and as restricted as possible. We established in the inpatient unit, and report here, some procedures, implemented in a dynamic way, to evaluate the adequacy of symptom-based surveys followed by screening tests for SARS-CoV-2 to patients with hematological diseases admitted in our unit, their relatives and health care personnel (5).

## Patients and Methods

Our Hematology Unit includes a six-isolated bed area for HSTC patients with positive pressure airflow rooms and a 22-bed area with filtered air for the regular management of patients with hematological disorders, mainly acute leukemias or aggressive non-Hodgkin Lymphoma, or treatment-related adverse events. In 2019, 138 transplants were performed (66 of them were allogeneic and 72, autologous) and 10 patients received infusion of CAR-T cells.

In response to the rapid virus spread in Salamanca, included within the region Castilla-León, the Hematology Department progressively developed an active surveillance plan focused on preventing the emergence of positive cases in our inpatient Unit, identifying suspected or confirmed COVID-19 cases between patients and staff members and establishing an action plan for when COVID-19 first case will appear.

### General Measurements

On March 9th, 7 days before the declaration of the National State of Alarm, we had an internal meeting to implement some measurements: (i) patients with planned chemotherapy treatments, autologous/allogeneic hematopoietic stem cell transplants or CAR-T cell infusions were carefully reviewed and all of them considered as non-urgent, were delayed, (ii) all visitors to the admission area were restricted, and (iii) all regular meetings for discussing patients and training activities were remotely scheduled (https://www.cdc.gov/coronavirus/2019-ncov/healthcare-facilities/prevent-spread-in-long-term-care-facilities.html) (6).

The access to our Unit was restricted for essential workers and patients were only taken out of the unit if it was strictly required.

About hygienic measures, thoroughly hands washing or repeated hydroalcoholic gel using were performed by staff members and properly surfaces and materials disinfecting were carried out; healthcare workers must wear a surgical mask what takes part of the routine in our Unit, and the utilization of FFP2 masks was mandatory later when they were available, disposable long-sleeved gown and gloves. In addition, for the regular visits from physicians, patients also wore a surgical mask (7, 8).

In case of a suspected or confirmed COVID-19 case, goggles or face shield and a long-sleeved water-resistant gown were added to this uniform (9). All symptomatic healthcare personnel with suspicion of COVID-19 infection were isolated at home until complete recovery and for at least 14 days. All asymptomatic staff members who had a direct contact with a positive case were considered as suspected COVID-19 case and equally isolated. Although caregivers were not allowed, in especial situations and when was needed, the same protocol for new inpatients was applied.

### Symptom and Radiologic Assessment

**Figure 1:**
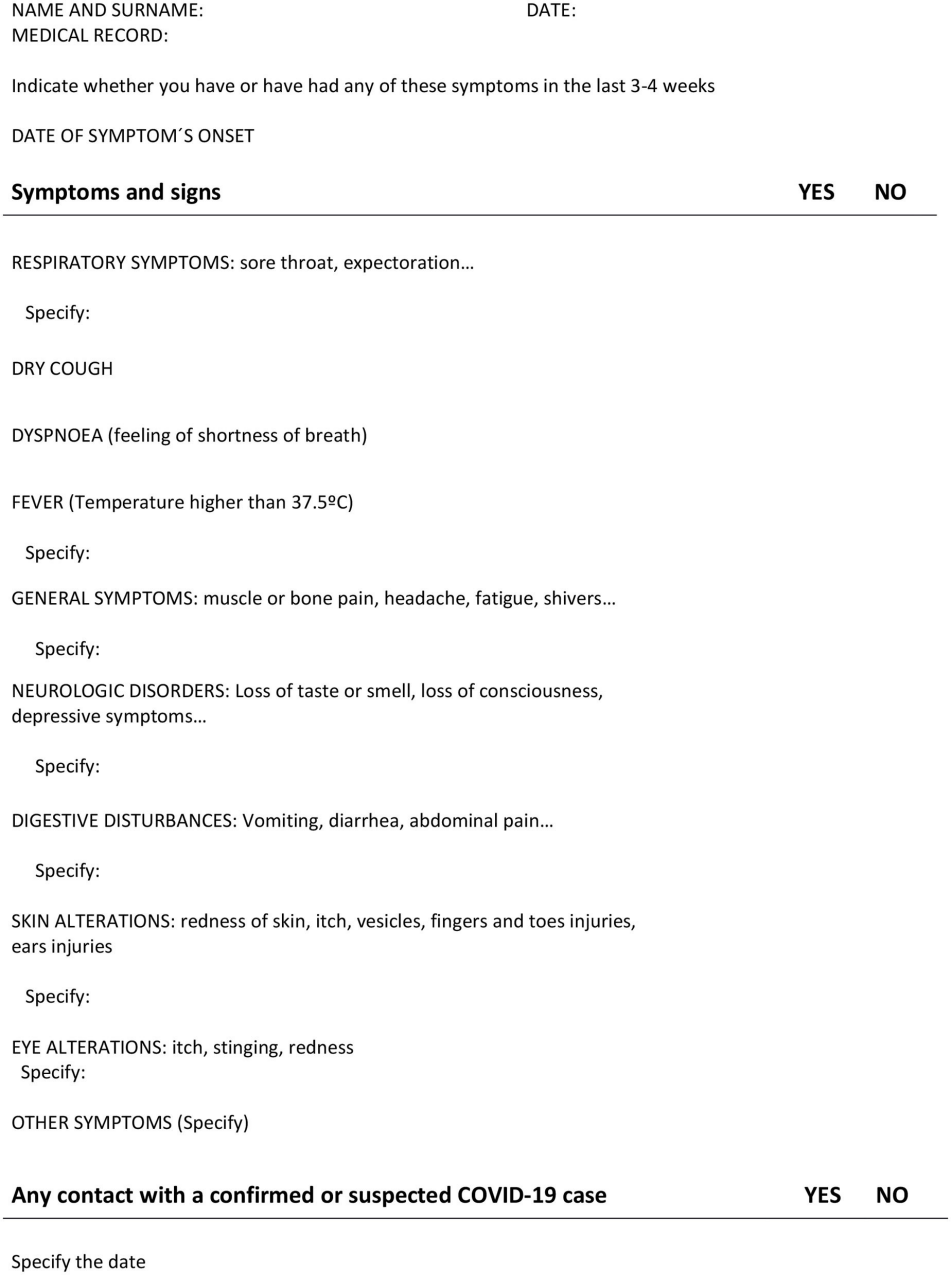
Standardized symptom-based form for screening.

A simple assessment form was performed, including questions about respiratory symptomatology and travel to cities with high incidence of COVID-19, and was completed for each patient that had been admitted at our Unit before March 9th and for each new patient who required hospitalization since this date until April 15th when we had the first case COVID-19 positive in the Unit and, based on the COVID-19-related systemic manifestations reported, we implemented the symptom-assessment form (Figure 1). In addition, for the new admissions, only asymptomatic patients, with no typical or atypical symptoms were allowed to be admitted in this area (10) and a chest X-ray was made to all of them as part of the baseline workout in our Unit.

### Laboratory Assessment

SARS-CoV-2 one-step real-time reverse transcriptase–polymerase chain reaction (rRT-PCR) diagnostic assay in a nasopharyngeal swab was performed for all suspected cases in our Unit, patients or healthcare personnel as well as for each new patient requiring admission to our Unit regardless of the symptomatology (11). The same protocol was applicable to the caregivers when they were necessary for the care of patients.

Symptomatic patients presenting with respiratory symptoms were tested for SARS-CoV-2 as well as for other respiratory virus; if the SARS-CoV-2 test was negative, the treatment or procedure was delayed and if hospitalization was required, they were admitted at another different area of the Hospital established for patients with clinical suspicion but negative rRT-PCR test. If the SARS-Cov-2 was positive, patients were admitted in the COVID-19 positive unit (12). If health care personnel resulted positive for SARS-CoV-2 infection, they were evaluated by the physicians responsible of the management of patients COVID-19 to proceed with the recommended work-up and decide the admission or isolation at home depending of the severity (13, 14).

In addition, when serological tests were available in our institution, all new admitted patients, caregivers, and healthcare personnel were also tested for antibodies and rT-PCR was subsequently performed only if serological tests was positive for IgG.

**Figure 2:**
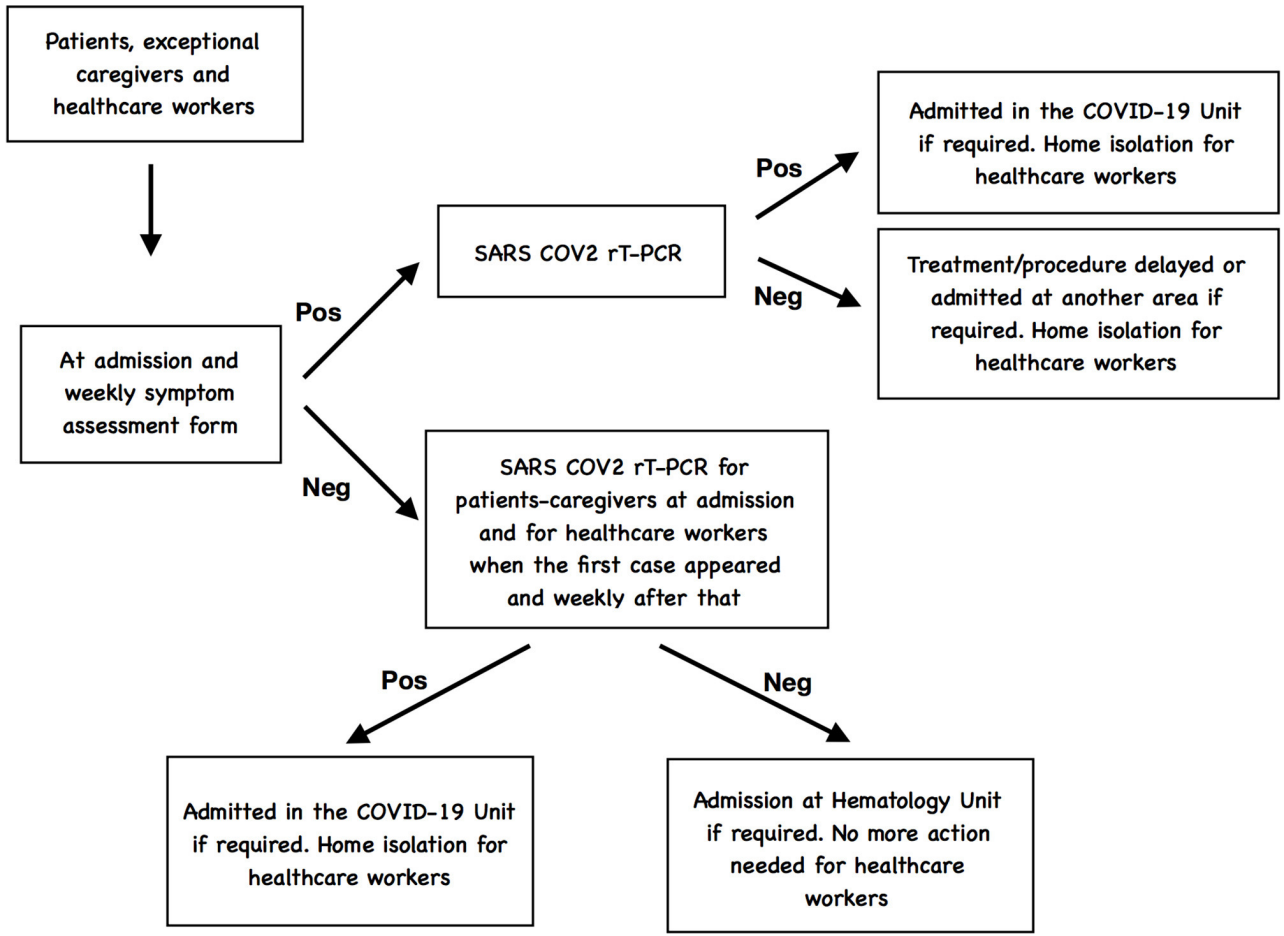
Flowchart-type diagram to represent the COVID-19 screening.

A flowchart-type diagram to represent the COVID-19 screening is showed in Figure 2.

The results are reported in a descriptive way so no statistical analysis has been performed. This study was conducted according to the Declaration of Helsinki and approved by the Ethics Committee of University Hospital of Salamanca.

## Results

On March 9th 2020, the inpatient situation in our Unit was: 14 patients in the regular hospitalization area (eight patients in the recovery process after transplant, autologous in four and allogeneic in the other four patients, and six patients receiving chemotherapy, or admitted because of treatment-related adverse events) and five patients in the HSCT Unit (two patients after CAR-T cells infusion and three patients in the earlier days after allogeneic stem cell transplantation).

On March 13nd 2020, when the national State of alarm was announced and according to our restrictions taken into consideration 7 days ago, nine patients had been discharged and scheduled chemotherapy or transplants non-urgent had been delayed. Notwithstanding, due to new admissions, 19 patients, finally, stayed in our Unit distributed as follows: 14 in the regular hospitalization area and the same five patients in the HSCT unit.

Between March 9th and April 28th and in spite of considering our measurements, 142 patients required admission into our inpatient Unit. The symptoms through the assessment forms were collected from all of them and the survey resulted positive in 78 patients (54.9%). They were considered as a suspected case and therefore admitted and isolated in a different Unit and managed by specific staff members. SARS-CoV-2 test was positive in 46 of them: This group of patients represents our population COVID-19 positive with hematological disorders admitted in our center in a specific unit and their outcome will be reported in a different manuscript.

The remaining 64 patients (45.1%) did not report any suspected symptomatology in the symptom-assessment form and represent the group of patients with hematological diseases admitted in our unit, between March 9th and April 28th, for which we planned our objective of maintaining them protected from SARS-CoV-2 infection. This group of patients required admission in our unit because of diagnosis of acute leukemia (five patients), scheduled chemotherapy (20 patients), autologous/allogeneic stem cell transplants that were not possible to be delayed (eight allogeneic and three autologous stem cell transplantation) and the rest were patients with treatment-related complications. Two additional patients received CAR-T cells infusions.

On April 15 a nursing assistant assigned at the HSCT Unit reported cough and fever at the end of her work shift. She was immediately considered as a suspected case and SARS-CoV-2 rRT-PCR confirmed the viral infection: she became the first positive case in our Unit. As consequence of that, our protocol was amended to perform screening tests for early identification of SARS-CoV-2 together with the collection symptoms in all inpatients, caregivers, and healthcare personnel.

The symptom-assessment form collected mild symptoms in just another nursing assistant becoming positive for SARS-CoV-2 infection. In addition, four additional healthcare personnel, two nurses and two nursing assistants became positive although the symptom-assessment forms were negative.

A 47 years old male with Ph+ Acute Lymphoblastic Leukemia newly diagnosed on April 8th, resulted positive for SARS-CoV-2 infection. He had not referred any symptomatology in the symptom-assessment form and the rRT-PCR for SARS-CoV-2 had previously been negative but he had been admitted at the HSCT unit (where the first COVID-19 nurse assistant was allocated); he was immediately isolated and moved to the COVID-19 Hematology Unit. The healthcare workers that were managing the patient were isolated at home and they did not report any symptomatology. The other two patients resulted negative and were moved into the general Hematology area and the HSCT unit was closed. The positive pressure and filtered air were turned-off. On the other hand, a thorough cleaning and fumigation were performed in the HSCT Unit.

On April 18, the only caregiver allowed to stay in our Unit, because of the mental status of her son, reported symptoms in the form and SARS-CoV-2 was positive and isolated at home. Her son was tested again and resulted negative. Nonetheless, the patient was considered as a direct contact, isolated, and managed as a suspected case in the COVID-19 Hematology Unit. Two subsequent rT-PCR tests were done every 2 days and after three successive assays as negative the specific measurements for COVID-19 isolation were lifted.

Of note, on April 17, two additional nurses who had been in direct contact with other positive staff members and whose first rT-PCR had been negative, reported atypical symptoms in the form and became positive. They were evaluated by the COVID-19 team at the Hospital and remained isolated at home.

**Figure 3:**
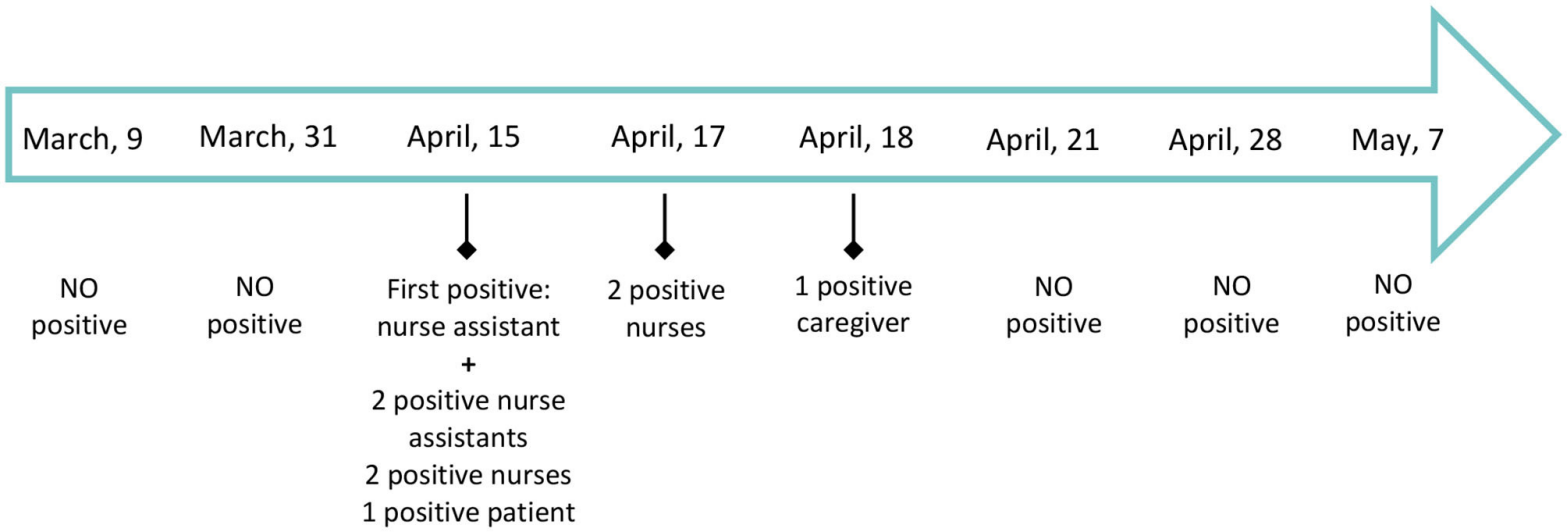
Timeline showing the positive COVID-19 cases in our Unit between March, 1st and May 7th, 2020. As April 28th, 2020, 32 patients and caregivers had been tested and a total of 68 rT-PCR diagnostic assays had been done: two positive cases were identified, only one of them with mild symptomatology. One hundred and six healthcare workers were tested with a total of 208 rT-PCR diagnostic assays: seven positive cases were identified of whom four presented any symptomatology. No more positive tests have been found since April, 18th.

On April 17, 48 h after its closure, the clinical work was resumed at the HSCT unit and the activity in the regular Hematology unit continued as previously. As a result of these measurements, 32 asymptomatic patients and caregivers in the inpatient unit had been tested and a total of 68 rT-PCR diagnostic assays had been performed to date with two positive results. One hundred and six healthcare workers had been tested, with a total of 208 rT-PCR diagnostic assays and seven of them were positive cases. The timeline showing the positive cases in our Unit is summarized in the Figure 3.

With the application of the actions previously mentioned, on April 28th, there has not been neither new patient nor health care worker reporting symptoms in the symptom-assessment form so the Hematology Unit has remained as a COVID-19 free unit.

In mid-May, serological tests were available in our institution and all admitted patients, caregivers, and healthcare personnel were tested to complete the COVID screening without new positive cases identified.

## Discussion

We report in this manuscript the strategy developed to maintain our Hematology Unit as a “COVID-19 negative island” in a hospital environment of positive areas with more than 600 patients admitted at the height of the pandemic. Salamanca is an university city with an incidence of 1,000 cases COVID-19 positive cases per 100,000 inhabitants (https://analisis.datosabiertos.jcyl.es/pages/coronavirus). The first consideration was the high rate of people infected by SARS-CoV-2 in Salamanca, in line with that reported in big cities like Madrid (1,046) or Barcelona (996), and some potential explanations are the 20,000 students Salamanca has as residents, its location in the central area of Spain, 212 Kilometers from Madrid as well as its touristic interest, receiving indeed many tourists from Madrid the week-end before the outbreak of COVID-19. Moreover, on March 8th it took place in Salamanca the final of the women's basketball cup chaired by the Queen of Spain with 4,368 attendees. The second consideration was the high number of inpatient cases tested in our Hospital, 1,584, resulting 1,100 of them positive for SARS-CoV-2. The peak of admissions occurred between March 21 and 31, with almost 600 patients. The result of these two considerations was our Hospital was basically converted into a COVID-19 hospital, the other specialties' activities reduced to the emergencies, oncology patients moved to another hospital and the Hematology Unit, because of its specific characteristics, remained as an island in the Hospital and our goal was to maintain this privileged condition. At the same time, our goal was to provide treatment to our patients in need, as safely and as justly as possible.

This was the rationale for the measures we decided to implement trying to early detect the suspected cases through the symptoms-reported assessment, doing screening tests to the patients, caregivers, and healthcare workers (15–17).

Concerning the triage assessment, our survey was implemented to be focused not only on respiratory symptoms and signs but included neurological, gastro-intestinal, skin, and ocular manifestations because of the new findings showing the SARS-CoV-2 infection is of systemic involvement (18, 19). Moreover, most patients with hematological disorders and recipients of transplants are receiving immunosuppressor agents and/or corticosteroids as concomitant treatment that could potentially modify the common presentation reported in immunocompetent population.

The accessibility for performing tests for early detection of SARS-CoV-2 infection has been an important limitation in our country and Hospital as well. In the general population, this fact is resulting on an underestimation of the number of real cases infected by SARS-CoV-2 but, in our Unit, we realized we were dealing with vulnerable and frail patients receiving intensive therapies including allogeneic stem cell transplantations with a curative approach. Therefore, as basis for crisis management, leadership, and strategies were developed to perform massive screening tests since April 15th where the nurse assistant become positive. This measurement was empirical because of the absence of national and international standardized protocols about the use of massive screening tests even in asymptomatic subjects. As April 28, 2020, 32 asymptomatic patients and caregivers in the inpatient unit were tested and a total of 68 rT-PCR diagnostic assays were done. One hundred and six healthcare workers were tested, with a total of 208 rT-PCR diagnostic assays. Apart from the positive cases presented on the results section, no more positive tests have been so far confirmed and we have been able to reschedule the activity delayed because of the outbreak.

Serological tests to evaluate the presence of antibodies were not available during this initial period (20) so they were later performed. At the time of writing this report, they are already available and planned to be performed although for patients with hematological disorders, the rT-PCR diagnostic assay should be the gold standard because of the immunosuppressive status that could potentially result in anergia and lack of immune response.

**Figure 4:**
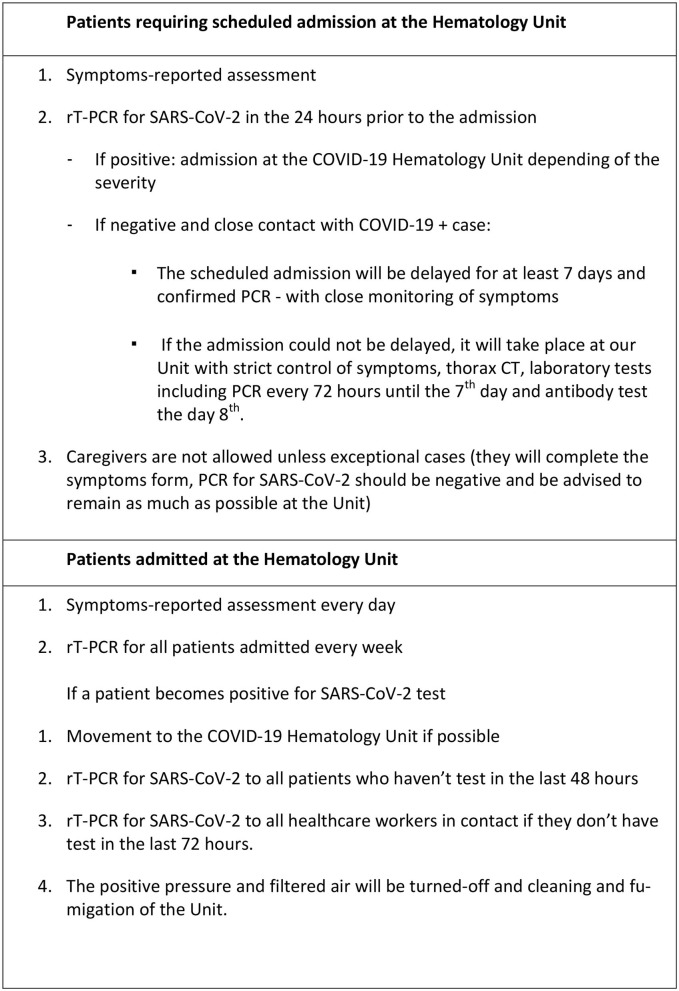
General measurements to maintain the Hematology Unit as a “COVID-19 free island” in the pandemic.

The outbreak of COVID-19 in Salamanca resulted in a brainstorming at the Hematology department to create strategies that would allow us to maintain our inpatient Hematology Unit as a “COVID-19 free island” and the Figure 4 represents the basic measurements implemented in our Unit that will remain active until the pandemic resolves.

In summary, based on the recognized vulnerability of Hematology units with the potential rapid and wide spread of the SARS-CoV-2, an appropriate protection with preemptive measures including a standardized symptom-assessment form and massive screening tests to all patients, caregivers, and healthcare personnel regularly, would be able to maintain COVID-19 free units providing adequate management to our immunocompromised patients.

## Data Availability Statement

All datasets generated for this study are included in the article/supplementary material.

## Author Contributions

M-DC, M-VM, and AC-M had the original idea and wrote the draft of the manuscript. AC-M collected the data of patients admitted in our unit. EP, AR, M-DC, and MG-D elaborated the first and amended versions of the local protocol for the management of patients in our Unit in the COVID-19 pandemic. FS-G, LL-C, AA, MB, MC, A-AM, BP, FP-M, L-GR, DP, LL-V, and M-BV involved in the management of patients in the Unit and have approved the manuscript. All authors contributed to the article and approved the submitted version.

## Conflict of Interest

M-VM has received honoraria from lectures and participation in advisory boards from Janssen, Celgene, Amgen, Takeda, GSK, Adaptive, Roche, Gennentech, Seattle Genetics, and Abbvie. FS-G has received lecturing and/or consulting honoraria in the last 3 years from Novartis, BMS, Pfizer, Incyte, Gilead, Roche, Amgen, and research support from Novartis. The remaining authors declare that the research was conducted in the absence of any commercial or financial relationships that could be construed as a potential conflict of interest.
